# *Srpk3* Decrease Associated with Alpha-Synuclein Increase in Muscles of MPTP-Induced Parkinson’s Disease Mice

**DOI:** 10.3390/ijms22179375

**Published:** 2021-08-29

**Authors:** Min Hyung Seo, Sujung Yeo

**Affiliations:** 1 Department of Meridian and Acupoint, College of Korean Medicine, Sang Ji University, Wonju 26339, Korea; cstcl@naver.com; 2 Research Institute of Korean Medicine, Sang Ji University, Wonju 26339, Korea

**Keywords:** Parkinson’s disease, srpk3, alpha-synuclein, MPTP, muscle

## Abstract

Parkinson’s disease (PD) is characterized by a loss of dopaminergic cells in the substantia nigra, and its histopathological features include the presence of fibrillar aggregates of α-synuclein (α-syn), which are called Lewy bodies and Lewy neurites. Lewy pathology has been identified not only in the brain but also in various tissues, including muscles. This study aimed to investigate the link between serine/arginine-rich protein specific kinase 3 (*srpk3*) and α-syn in muscles in PD. We conducted experiments on the quadriceps femoris of a 1-methyl-4-phenyl-1,2,3,6-tetrahydropyridine (MPTP)-induced PD mouse model and the C2C12 cell line after treatment with 1-methyl-4-phenylpyridinium (MPP+) and *srpk3* short interfering RNA (siRNA). Compared to the control group, the MPTP group showed significantly reduced expression of srpk3, but increased expression of α-syn. In MPP+-treated C2C12 cells, srpk3 expression gradually decreased and α-syn expression increased with the increasing MPP+ concentration. Moreover, experiments in C2C12 cells using srpk3 siRNA showed increased expressions of α-syn and phosphorylated α-syn. Our results showed that srpk3 expression could be altered by MPTP intoxication in muscles, and this change may be related to changes in α-syn expression. Furthermore, this study could contribute to advancement of research on the mechanism by which srpk3 plays a role in PD.

## 1. Introduction

Parkinson’s disease (PD) is a neurodegenerative disorder characterized by dopaminergic cell loss in the substantia nigra (SN). The main symptoms of PD are motor dysfunction, including bradykinesia, tremor, muscle rigidity, postural instability, and walking impairments. PD usually occurs in elderly people and can lead to dangerous events, such as falls, which can lead to secondary injuries. In addition, dyskinesia could be due to insufficient muscle recruitment for the initiation of movement [1].

The histopathological features of PD include the presence of fibrillar aggregates of α-synuclein (α-syn), which are called Lewy bodies and Lewy neurites. Lewy pathology has been identified not only in the brain but also in various tissues, including the enteric nervous system [2,3,4], cardiomyocytes [5,6,7], skin [8,9] and muscles [10,11]. Exercise improves motor function in patients with PD [12,13]. Consistent exercise and engaging in regular exercise are associated with significant positive effects on health-related quality of life and motor function changes [14]. High-intensity treadmill exercise is also feasible and safe for patients with PD [15]. The study of aerobic exercise also showed generic health benefits for people with PD, including a reduced incidence of mortality and cardiovascular disease and improved bone health [16]. These studies suggest a link between muscle-related factors and PD.

Serine/arginine-rich protein specific kinase 3 (*srpk3*) belongs to the serine/arginine protein kinase family and is controlled by a muscle-specific enhancer regulated by mef2 [17]; srpk3 is expressed in the lungs, skin, spleen, heart, joints, brain, and muscles. This gene encodes a protein kinase that is specific for the serine/arginine-rich domain family of splicing factors, which phosphorylates serine/arginine repeat-containing proteins [17]. This protein plays an important role in muscles [17].

Since the main symptoms of patients with PD are motor function-related symptoms and the symptoms improve with exercise, we deemed that muscle or motor function-related factors might be involved. Therefore, in the current study, we investigated the expression of srpk3 in the muscles of a 1-methyl-4-phenyl-1,2,3,6-tetrahydropyridine (MPTP)-induced parkinsonism mouse model [18]. The C2C12 cell line was used to determine changes in α-syn expression following changes in *srpk3* expression.

We hypothesized that srpk3 expression can be altered by MPTP intoxication in muscles, and this change would be related to changes in α-syn expression. The purpose of this study was to investigate the link between srpk3 and α-syn in muscles in a PD mouse model. Furthermore, this study could contribute to advancing research on the mechanism of srpk3 as an element related to PD.

## 2. Results

**Figure 1:**
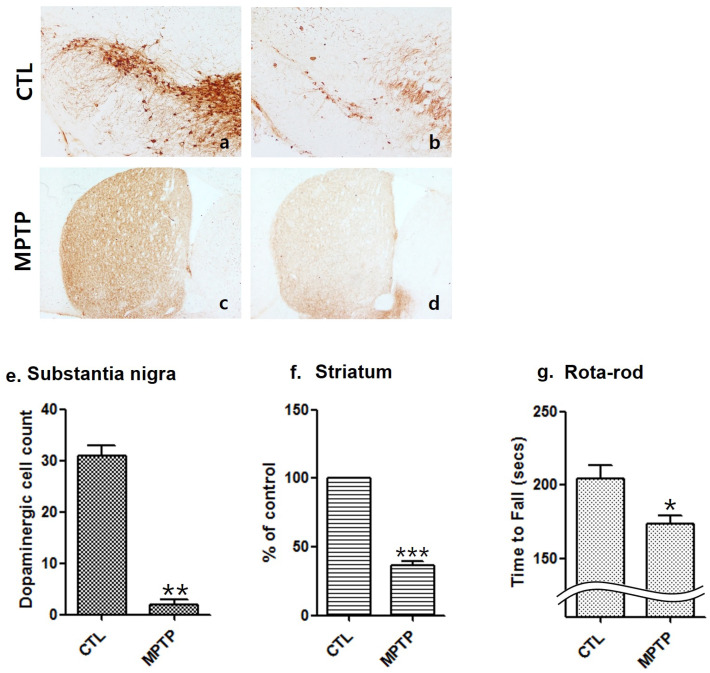
Reduction of dopaminergic cells and tyrosine hydroxylase expression in an MPTP-induced Parkinson’s disease mouse model. Immunohistochemically stained substantia nigra (SN, (**a**,**b**)) and striatum (ST, (**c**,**d**)) using a tyrosine hydroxylase (TH; (**a**,**b**), 100×; (**c**,**d**), 40×) antibody. (**e**) Decrease in dopaminergic cells in the SN in the MPTP group (*n* = 3). (**f**) Immunostaining analyses of the TH level per area in the ST showing the decrease in TH expression in the MPTP groups (*n* = 3). (**g**) The rotarod (28 mm diameter) test was conducted during 5 min of running time. First, the Accel Forward mode from 10 to 50 rpm was performed for 4 min and a constant speed of 50 rpm maintained in the final 1 min (*n* = 6). CTL, control (phosphate-buffered saline); MPTP, 1-methyl-4-phenyl-1,2,3,6-tetrahydropyridine. (* *p* < 0.05, ** *p* < 0.01, and *** *p* < 0.0005 were compared to control).

To confirm the MPTP-induced Parkinson’s disease mouse model, the SN (Figure 1a,b) and striatum (ST) (Figure 1b,d) regions were immunohistochemically stained with tyrosine hydroxylase (TH). Compared to the control (CTL) group, the MPTP group showed decreased dopaminergic neurons in the SN (Figure 1e) and TH expression in the ST (Figure 1f). Consistent with the immunohistochemistry results, the rotarod test also demonstrated a shorter running time in the MPTP group than in the CTL group (Figure 1g).

**Figure 2:**
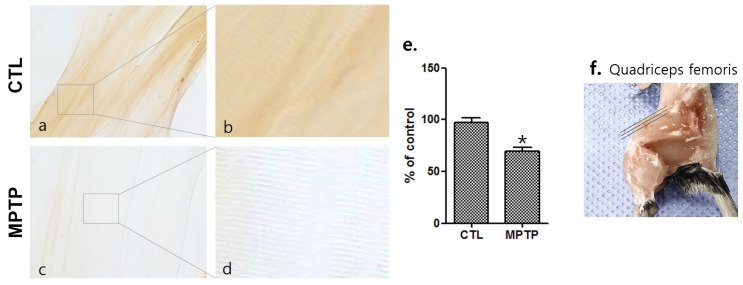
Decrease in serine/arginine-rich protein specific kinase 3 (*srpk3*) expression in the quadriceps femoris of a chronic MPTP-induced Parkinson’s disease mouse model. Immunohistochemically stained quadriceps femoris using anti-srpk3 antibody ((**a**,**c**), 400×; (**b**,**d**), magnified images of square). (**e**) Histograms of the immunohistochemistry showing the decrease in *srpk3* expression in the MPTP group, compared to CTL group, in which the expression was normalized to 100% (*n* = 3). (**f**) An image of the quadriceps femoris of C57BL/6 mouse. The three lines indicate the cutting direction. CTL, control (phosphate-buffered saline); MPTP, 1-methyl-4-phenyl-1,2,3,6-tetrahydropyridine. (* *p* < 0.01 was compared to control).

To investigate the change in the srpk3 expression in the skeletal muscle of the MPTP-induced Parkinson’s disease mouse model, the quadriceps femoris was immunohistochemically stained using anti-srpk3 antibody (Figure 2). Compared to the CTL group, the MPTP group had significantly reduced srpk3 expression (*p* < 0.01).

**Figure 3:**
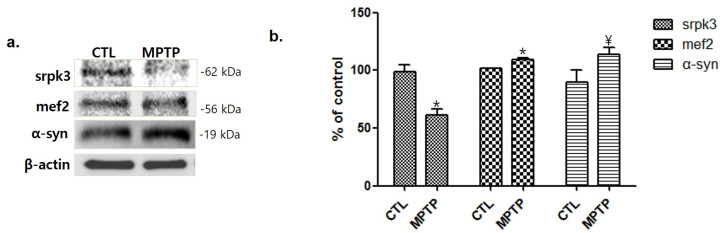
Decrease in serine/arginine-rich protein specific kinase 3 (*srpk3*) expression in the quadriceps femoris of an MPTP-induced parkinsonism mouse model. (**a**) The results of the Western blot are shown. (**b**) Histograms of the Western blot analyses showing decreased srpk3 expression and increased mef2 and α-synuclein expression in the MPTP group compared to the CTL group, in which the expression was normalized to 100% (*n* = 3). CTL, control (phosphate-buffered saline); MPTP, 1-methyl-4-phenyl-1,2,3,6-tetrahydropyridine. (* *p* < 0.05 and ¥ = 0.18 were compared to CTL).

Consistent with the results of immunohistochemistry, Western blotting also showed a decreased srpk3 expression in the MPTP group compared to that in the CTL group (*p* < 0.05). The expression of mef2, which is a muscle-specific enhancer controlling srpk3, and α-syn increased in the MPTP group compared to that in the CTL (Figure 3).

**Figure 4:**
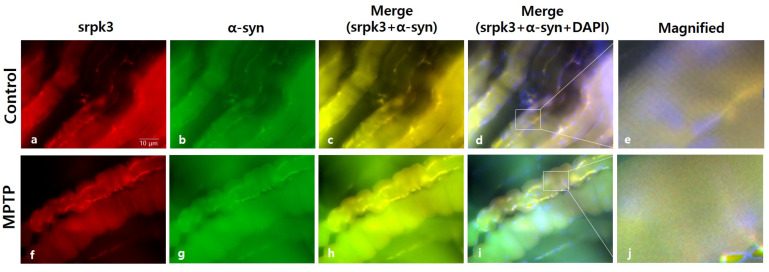
Immunofluorescence staining of serine/arginine-rich protein specific kinase 3 (*srpk3*) and α-synuclein (α-syn) in the quadriceps femoris of an MPTP-induced parkinsonism mouse model. Control group: (**a**) srpk3; (**b**) α-syn; (**c**) merging of (**a**,**b**); (**d**) merging of (**a**,**b**), and DAPI (nuclei); (**e**) magnified image of the square. MPTP group: (**f**) srpk3; (**g**) α-syn; (**h**) merging of (**f**,**g**); (**i**) merging of (**f**,**g**), and DAPI; (**j**) magnified image of the square (scale bar, 10 µm).

We conducted immunofluorescence staining of srpk3 and α-syn in the quadriceps femoris to confirm the altered expression induced by MPTP. In the MPTP group, the merging of srpk3 and α-syn decreased (Figure 4c,h), and muscle streaks were reduced (Figure 4e,j).

**Figure 5:**
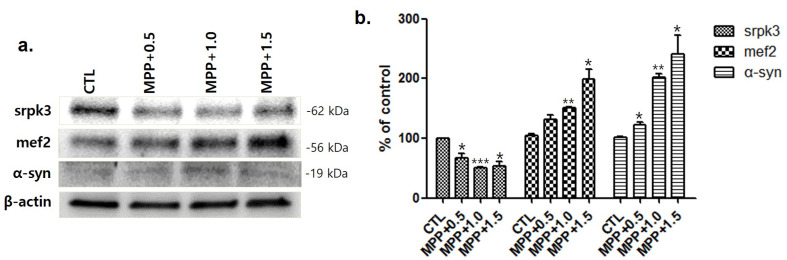
Western blot analysis of components linked to serine/arginine-rich protein specific kinase 3 (*srpk3*) at different 1-methyl-4-phenylpyridinium (MPP+) concentrations in C2C12 cells. (**a**) Western blot analysis of *srpk3*, mef2, and α-synuclein (α-syn) at different MPP+ concentrations treated: 0.5, 1.0, and 1.5 mM. MPP+ was applied for 18 h. (**b**) Quantified protein levels of the Western blot results (*n* = 3). (* *p* < 0.05, ** *p* < 0.01, and *** *p* < 0.005 were compared to control).

To investigate the relationship between srpk3 and α-syn expression, we conducted an experiment in 1-methyl-4-phenylpyridinium (MPP+)-treated C2C12 cells. As the MPP+ concentration increased, srpk3 expression gradually decreased; however, mef2 and α-syn expression increased (Figure 5).

**Figure 6:**
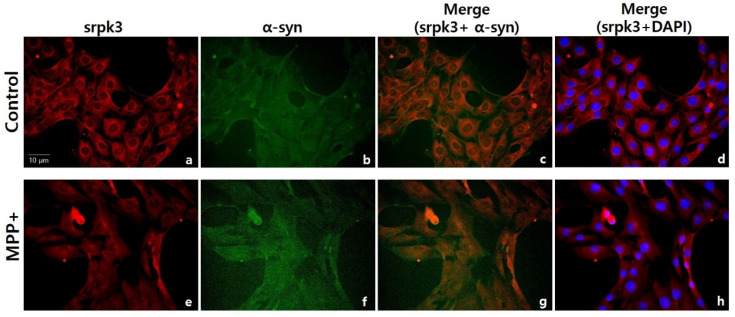
Immunofluorescence staining of serine/arginine-rich protein specific kinase 3 (*srpk3*) and α-synuclein (α-syn) in C2C12 cells. Control group: (**a**), srpk3; (**b**) α-syn; (**c**) merging of (**a**,**b**); and(**d**) merging of a and DAPI (nuclei). 1-methyl-4-phenylpyridinium (MPP+) group: (**e**) srpk3; (**f**) α-syn; (**g**) merging of (**e**,**f**); and (**h**) merging of (**e**) and DAPI (scale bar, 10 µm).

On immunofluorescence staining of *srpk3* and α-syn in MPP+-treated C2C12 cells, the CTL group showed a higher density of srpk3 expression than the MPP+ group and srpk3 expression around the nucleus was thicker than that in other regions. However, the MPP+ group showed a weak expression of srpk3 and increased expression of α-syn compared to CTL group (Figure 6).

**Figure 7:**
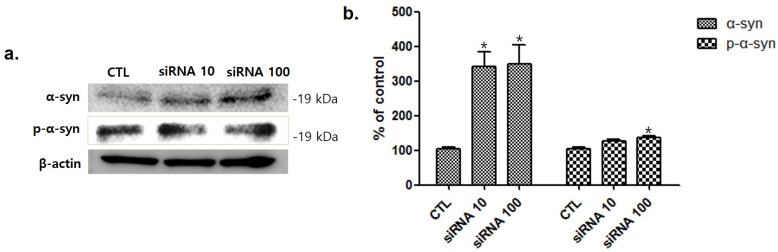
Western blot analysis of α-synuclein (α-syn) and phosphorylated-α-syn (p-α-syn) after treatment of C2C12 cells with serine/arginine-rich protein specific kinase 3 (*srpk3*) siRNA. (**a**) Western blot assay of α-syn and p-α-syn, after *srpk3* siRNA application. (**b**) Quantified protein levels of the Western blot results (*n* = 3). Control (CTL), negative control siRNA treatment (100 nM for 2 days); siRNA 10, *srpk3* siRNA treatment (10 nM for 2 days); siRNA 100, *srpk3* siRNA treatment (100 nM for 2 days). (* *p* < 0.05 was compared to control).

To investigate the relationship between srpk3 and α-syn expression, we conducted an experiment in C2C12 cells using srpk3 siRNA. As cell lines were treated with *srpk3* siRNA, which reduces *srpk3* expression, α-syn and phosphorylated-α-syn (p-α-syn) expression increased (Figure 7).

## 3. Discussion

We confirmed that compared to the CTL group, the MPTP group showed decreased dopaminergic neurons in the SN and TH expression in the ST. The rotarod test demonstrated shorter running time in the MPTP group than in the CTL group.

In these mice, we investigated the change in srpk3 expression in the skeletal muscle quadriceps femoris. The results showed that srpk3 expression was significantly reduced in the MPTP group compared to that in the CTL group. In addition, our data demonstrated an increased mef2, which is a muscle-specific enhancer controlling srpk3, and α-syn expression in the MPTP group compared to that in the CTL group. In the immunofluorescence staining of srpk3 and α-syn in the quadriceps femoris, we confirmed the altered expression induced by MPTP, which showed a decreased merging of srpk3 and α-syn and reduced muscle streaks in the MPTP group.

As the MPP+ concentration increased in MPP+-induced C2C12 cells, srpk3 expression gradually decreased, but the expression of mef2 and α-syn increased in the MPTP group compared to that in the CTL group. As a transcription factor, mef2 plays a role in various tissues, including muscles. Altered mef2 expression has recently been reported to be associated with several cancers and plays a role in human diseases [19]. The activities of mef2 are related to the regulation of cytoskeletal and neuronal cytoskeletal structures [20,21]. In addition, *srpk3* is controlled by a muscle-specific enhancer regulated by mef2 [17]. Our results showed an increased expression of mef2 in the MPTP parkinsonism mouse and MPP+-treated C2C12 cells, whereas srpk2 expression was decreased in these models. This suggests that the decrease in srpk3 in parkinsonism is not caused by mef2 and that the decrease in srpk3 expression could be caused by MPTP or MPP+, which induced parkinsonism. Increased expression of mef2 is thought to compensate for the decreased expression of srpk3.

In immunofluorescence staining of *srpk3* and α-syn in MPP+-treated C2C12 cells, the CTL group demonstrated a higher density of srpk3 expression than the MPP+ group, and srpk3 expression around the nucleus was thicker than that in other regions. However, the MPP+ group showed a weak expression of srpk3 and increased expression of α-syn compared to CTL group (Figure 5).

To investigate the relationship between srpk3 and α-syn expression, we conducted an experiment in C2C12 cells using srpk3 siRNA, which reduces srpk3 expression. As cell lines were treated with siRNA against srpk3, α-syn and p-α-syn expression gradually increased. This suggests that srpk3 reduction can cause changes in α-syn expression.

Pathological changes in α-syn have been identified in various tissues including muscles [10,11], and recent research has shown that motor function-related symptoms could be associated with neuro-muscular problems related to α-syn [22]. Consistent with this, our results demonstrated an increase α-syn expression in the muscles in the MPTP group compared to that in the CTL group. Our results showed srpk3 reduction in the quadriceps femoris in the MPTP group, which was thought to be related to pathological changes in α-syn. In an experiment in C2C12 cells using srpk3 siRNA, increased expression of α-syn and p-α-syn was observed. These results suggest that srpk3 expression can be associated with α-syn expression and that increased expression of α-syn induced by srpk3 downregulation might be involved in pathological changes in muscles of patients with PD.

Recently, the concept of interneuronal spread of α-syn misfolding aggregates from the peripheral nervous system to the brain, via the enteric or sensory nervous systems, has been reported [23]. The prion-like propagation of α-syn is now widely accepted in the field and is the subject of intense research [24,25]. Based on these previous studies, we propose that the increased expression of α-syn induced by srpk3 downregulation in the muscle might be one of the causes of the spread of α-syn.

The MPTP model has several major flaws and does not recapitulate the cardinal features of PD, such as the Lewy body pathology and hyperphosphorylation of α-syn. Therefore, all potential findings obtained with the current MPTP model need to be taken with a certain degree of caution.

## 4. Materials and Methods

### 4.1. MPTP-Induced PD Mouse Model

To produce a semi-chronic model of PD, 6-week-old male C57BL/6 mice (20–22 g; DBL, Eumseong-gun, Korea) were divided into two groups—the CTL and MPTP groups. In the CTL group, mice were injected intraperitoneally with 100 μL phosphate-buffered saline (PBS), while in the MPTP group, mice were injected intraperitoneally with MPTP-HCL (20 mg/kg of free base; Sigma, St. Louis, MO, USA) in PBS (100 μL) every 24 h for 4 weeks. On the day after the final MPTP treatment, mice were anesthetized using Alfaxan and perfused transcardially with cold PBS. All animal experiments in this study were approved by the Sang Ji University Animal Experimentation Committee (approval code: 2016-07; date of approval 29 March 2016).

### 4.2. Rotarod Test

Before the last injection, a rotarod test was performed to evaluate the motor ability. Training was performed at 30 rpm for 15 min, once a day in the second week for 2 days. The rotarod treadmill diameter was 280 mm, and the rotarod (28 mm diameter) test was conducted during 5 min of running time. First, the Accel Forward mode from 10 to 50 rpm was performed for 4 min and a constant speed of 50 rpm was maintained in the final 1 min. The time until the first fall was scored.

### 4.3. Immunohistochemistry

After the mice were sequentially perfused with PBS and 4% paraformaldehyde, the tissues were fixed in 4% paraformaldehyde for 1 day at 4 °C and dehydrated in 30% sucrose buffer for 3 days at 4 °C. The sections (40 μm) were cut using a cryomicrotome. The sections encompassing the ST and SN regions were incubated in 3% H_2_O_2_ with PBS (pH 7.4) and then blocked in PBS containing 1% bovine serum albumin (BSA) and 10% horse serum. After treatment with an M.O.M mouse Ig-blocking reagent (Vector Laboratories, Burlington, ON, Canada) for 1 h at room temperature, the sections were incubated with the primary antibody overnight at 4 °C. Thereafter, the sections were treated with a biotinylated anti-mouse IgG and an avidin–biotin–peroxidase complex.

### 4.4. Cell Lines and Cultures

C2C12 cells were incubated in 5% CO_2_ at 37 °C in Dulbecco’s modified Eagle’s medium (BioWest, Riverside, MO, USA) containing 10% fetal bovine serum (GenDEPOT, Katy, TX, USA) and 100 U/mL penicillin–streptomycin (Gibco, Amarillo, TX, USA).

### 4.5. MPP+ Treatment

C2C12 cells were treated with 0.5, 1, or 1.5 mM MPP+ iodide (Sigma) for 18 h. MPP+ administration was performed simultaneously in each experiment.

### 4.6. Short Interfering RNA Knockdown

C2C12 cells were cultured in Opti-MEM medium (Gibco, Amarillo, TX, USA) and then transfected with siRNA for 48 h. When the density of C2C12 cells was 30%, transfection reagent and srpk3 siRNA (3.5:1) were applied; siRNA against *srpk3* (5-GAA AAC UGC CUG UUU GUU U-3) and negative control duplexes (5-UUC UCC GAA CGU GUC ACG UTT-3) were used (Bioneer Inc., Daejeon, Korea).

### 4.7. Western Blotting

The quadriceps femoris muscle tissues were homogenized in 20 mM radioimmunoprecipitation assay buffer on ice for 20 min using a sonicator (Qsonica Q55, Newtown, CT, USA). C2C12 cells were homogenized in Tris-Triton cell lysis buffer (GenDEPOT) for 20 min on ice.

After centrifugation of the tissues and the C2C12 cells at 12,000 rpm at 4 °C for 15 min, supernatant samples were separated using sodium dodecyl sulfate-polyacrylamide gel electrophoresis. The gel was transferred to a polyvinylidene difluoride membrane (Pall Life Science, New York, NY, USA), and the membrane was blocked with 3% BSA at room temperature. After blocking, the membrane was incubated with anti-α-syn (1:500; Santa Cruz Biotechnology, Dallas, TX, USA), anti-srpk3 (1:1000; Cloud-Clone Corp., Katy, TX, USA), or anti-β-actin (1:5000, Santa Cruz Biotechnology, Dallas, TX, USA) antibodies overnight and washed with 0.1% Tris-buffered saline containing Tween 20 (TBST). The membrane was then incubated with a secondary antibody for 1 h at room temperature and washed with 0.1% TBST.

### 4.8. Immunofluorescence

Longitudinal quadriceps femoris muscle cryosections were fixed in 4% paraformaldehyde and methanol. After fixation, sections were blocked in PBS with 1% BSA and 5% goat serum for 1 h. C2C12 cells were fixed in 4% paraformaldehyde and blocked for 1 h. The samples were incubated with anti-α-syn (1:500) and anti-srpk3 (1:1000), and then incubated with secondary antibodies, goat anti-mouse IgG (H + L) fluorescein isothiocyanate-conjugated (CUSABIO, Houston, TX, USA), and goat anti-rabbit IgG (H + L) tetramethylrhodamine-conjugated (Invitrogen, Carlsbad, CA, USA). Finally, DAPI was used to label the cell nuclei (1 μg/mL). Photographic documentation was obtained using a Nikon X-cite series 120Q microscope (Nikon, Tokyo, Japan). The exposure parameters were the same for each group.

### 4.9. Imaging Software

ImageJ software 1.53e (University of Wisconsin, Madison, WI, USA) was used for the images analysis.

### 4.10. Statistical Analysis

Statistical analysis was performed using Student’s *t*-test and analysis of variance in SPSS 25 (SPSS Inc., Released 2017, PASW Statistics for Windows, Version 25.0, Chicago, IL, USA). All values show mean ± standard error.

## 5. Conclusions

In conclusion, in this study, we studied the link between srpk3 and α-syn in the muscles of an MPTP-induced PD mouse model. Our results showed that srpk3 expression could be altered by MPTP intoxication in muscles, and this change may be related to changes in α-syn expression. Moreover, this study could contribute to advancing research on the mechanism of srpk3 as an element related to PD.

## Data Availability

All data analyzed during this study are included in this published article.
